# Improving Cell Viability and Velocity in μ-Extrusion Bioprinting with a Novel Pre-Incubator Bioprinter and a Standard FDM 3D Printing Nozzle

**DOI:** 10.3390/ma14113100

**Published:** 2021-06-05

**Authors:** Juan C. Gómez-Blanco, Victor Galván-Chacón, David Patrocinio, Manuel Matamoros, Álvaro J. Sánchez-Ortega, Alfonso C. Marcos, María Duarte-León, Federica Marinaro, José B. Pagador, Francisco M. Sánchez-Margallo

**Affiliations:** 1 Jesús Usón Minimally Invasive Surgery Centre, 10071 Cáceres, Spain; vpgalvan@ccmijesususon.com (V.G.-C.); dpatrocinio@ccmijesususon.com (D.P.); mduarte@ccmijesususon.com (M.D.-L.); fmarinaro@ccmijesususon.com (F.M.); msanchez@ccmijesususon.com (F.M.S.-M.); 2 School of Industrial Engineering, University of Extremadura, 06006 Badajoz, Spain; manuelmp@unex.es (M.M.); ajs@unex.es (Á.J.S.-O.); acmarcos@unex.es (A.C.M.)

**Keywords:** bioprinting, pre-incubator, atmospheric enclosure, 3D printing, nozzle, conical tip, fluid flow, cell viability

## Abstract

Bioprinting is a promising emerging technology. It has been widely studied by the scientific community for the possibility to create transplantable artificial tissues, with minimal risk to the patient. Although the biomaterials and cells to be used are being carefully studied, there is still a long way to go before a bioprinter can easily and quickly produce printings without harmful effects on the cells. In this sense, we have developed a new μ-extrusion bioprinter formed by an Atom Proton 3D printer, an atmospheric enclosure and a new extrusion-head capable to increment usual printing velocity. Hence, this work has two main objectives. First, to experimentally study the accuracy and precision. Secondly, to study the influence of flow rates on cellular viability using this novel μ-extrusion bioprinter in combination with a standard FDM 3D printing nozzle. Our results show an X, Y and Z axis movement accuracy under 17 μm with a precision around 12 μm while the extruder values are under 5 and 7 μm, respectively. Additionally, the cell viability obtained from different volumetric flow tests varies from 70 to 90%. So, the proposed bioprinter and nozzle can control the atmospheric conditions and increase the volumetric flow speeding up the bioprinting process without compromising the cell viability.

## 1. Introduction

Bioprinting is nowadays an emerging technology widely used in regenerative medicine and tissue engineering. It can be defined as a technology capable of an accurate deposition of cells and biochemical components layer by layer to create defined structures using materials, bioactive molecules and cells [1,2]. The creation of artificial tissues and organs using patient cells can reduce the rejection risk [3]. Among all possible tissues to recreate, research is focused in bioprinting vascular, neural, bone, cardiac, skin or muscle tissues [4,5]. To obtain a fully functional tissue all properties and functionalities of the desired tissue must be considered and adapted to the material and cell-line for the bioprinting procedure. In this sense, not only the bioink must be appropriate for the process but also the bioprinting procedure itself. The most common technologies in bioprinting are μ-extrusion, inkjet, laser-assisted and stereolithography [6]. Independently of the bioprinting procedure the ideal bioprinter was described by Dababneh and Ozbolat [7] and they concluded that this bioprinter must have 10 main characteristics:

- High degree-of-freedom in motion.
- High resolution and accuracy.
- The ability to dispense various bioink solutions simultaneously.
- Ease of use.
- Compact size.
- Ease of sterilization.
- Full-automation capability.
- Affordability.
- Versatility.

Current bioprinters can fulfill some of the ideal bioprinter characteristics but they are highly influenced by the technology they used. Heinrich et al. [5] performed a review of the most used bioprinting techniques. From this data, it can be obtained that the most relevant advantages are the possibility of using high viscous bioinks with high cell density as well as their simple process for μ-extrusion; the accurately deposit a small number of cells in a fast process with high cell viability (80–90%) for inkjet; or high cell viability (up to 95%) for laser-assisted bioprinting [5,6]. Additionally, the main disadvantages of the previous bioprinting techniques are the relative low printing speed with moderate cell viability (40–80%) for μ-extrusion and the limited bioink and cell density for inkjet, as well as the complexity of the system for laser-assisted bioprinting. Despite being a slow procedure with moderate cell viability, μ-extrusion bioprinting is the most used because it is highly versatile and brings the possibility of using a wide range of bioink viscosities and cells densities [3,6,8]. This high versatility of the μ-extrusion bioprinting is also dependent of the bioink used. Hydrogels, as the most used cell-laden material, must have specific properties to be fully suitable to bioprinting. According to bibliography, alginate is one of the most used components in cell laden bioprinting along with gelatin and GelMA [9]. In this sense, alginate is widely used in many sorts of biomedical applications as bone or cartilage regeneration, cardiovascular system formation or other tissues regeneration [10]. The possible applications of alginate makes this material to be widely studied. Many authors are trying to enhance its poor mechanical properties while maintaining or increasing its good biocompatible properties [11]. As an example, Pan et al. [12] are trying to enhance the cell viability by encapsulating the cells using an alginate hydrogel blend with pyrogallol. This approach obtained a good cytoprotection of the cells while they can modulate cell distribution as well as cell migration and proliferation. Wei et al. [13] have improved the printability of an alginate and gelatin hydrogel with the addition of bioactive glass nanoparticles. They obtained a proper hydrogel to extrude enhancing the proliferation and cell adhesion without changing the porous structure of the alginate or the biodegradation rate of the hydrogel.

Regarding μ-extrusion bioprinters, there are a wide variety of commercial bioprinters in the market. Among the most affordable ones (less than 20,000 $), compact size and high resolution and accuracy are usually common features within a resolution range between 50 and 200 μm [14]. More expensive bioprinters or laboratory non-commercial bioprinters have better resolution, accuracy and printing speed. Independently of the affordability of the bioprinter, some of them have enclosure systems capable of controlling the air flow through High Efficiency Particle Arresting (HEPA) filters, such as Cellink BioX^®^ (Cellink; Boston, MA, USA), Poietis^®^ (Poietis; Pessac, France) or 3D-Discovery BioFactory^®^ (REGENHU; Villaz-Saint-Pierre, Switzerland) [15,16,17]. As far as authors know, only Matamoros et al. [18] have developed and tested an atmospheric enclosure (pre-incubator) capable of controlling the air flow as well as the inner temperature and humidity. Their system can be considered as a pre-incubator, preventing bioink to dry and cells to have better environment to increase their viability. Regarding to the possibility of dispensing various bioinks simultaneously, many bioprinters have multiple print-heads and a few have also rapid interchangeable print-heads as the Cellink BioX^®^ (Cellink; Boston, MA, USA) [15]. Furthermore, most of μ-extrusion bioprinters use a pneumatic approach to perform the extrusion of the material. This approach makes the bioprinter much easier to be used but it neglects the control of volumetric flow, which might reduce the shape fidelity of the printed tissue [3,6,8].

Current research on μ-extrusion bioprinting mechanics is focused on two pathways, minimizing the costs and obtaining the best shape fidelity by using piston driven extrusion. In this sense, authors have been developing and adapting new piston-driven extrusion-head for conventional 3D printers [19,20,21,22,23,24] or developing new bioprinters [25,26]. New adapted extrusion-heads are usually based on a design proposed by Wijnen et al. [27]. This design is open-source, easily adaptable to any 3D printer and mainly composed by a NEMA motor, a worm screw, a syringe holder and a syringe pusher. Nevertheless, large sizes and weights of extrusion-head have been related to low precision and accuracy of the system [27]. Kahl et al. [23] modified the initial design by changing the location of the motor and reducing possible extruder-head movements by inertia. Regarding to the creation of new bioprinters, Campbell et al. [25] used the same extruder head proposed by Wijnen et al. [27] but using external reservoirs connected to a new extruder-head through heated/cooled tubes. The bioprinter designed by McElheny et al. [26] used the same open source extruder but the NEMA motor was changed by a noncaptive stepper motor. Additionally, authors as Ravi et al. [28] or Yenilmez et al. [29] developed and tested new bioprinting systems combining inkjet, FDM and μ-extrusion; and inkjet, μ-extrusion and UV light, respectively.

Not only bioprinters and extrusion heads are in the focus on μ-extrusion bioprinting improvement but also the nozzles. The internal geometry of the nozzle modifies inner bioprinting parameters such as pressure, velocity or shear stress which might have an important impact on the features of the bioprinted structures [30]. In this sense, computational simulation is commonly used to study inner bioprinting parameters [24,31,32,33,34,35,36] mainly due to the difficulty to perform experimental measurements without disturbing the flow of bioinks. Specifically, Reid et al. [24], Gómez-Blanco et al. [31] or Martanto et al. [35] demonstrate that different conical tips gauges have a noticeable influence in the volumetric flow and the shear stress using numerical methods or computational simulations. Additionally, in a recent work Gómez-Blanco et al. [37] demonstrated that the use of a 3D printer standard nozzle can increase the velocity of the process when compared with a conical tip. The proposed method was also shown to have a reduced impact on pressure and shear stress that should keep similar levels of cellular viability. In this sense, this achievement can modify the actual limitation of low velocity of μ-extrusion bioprinting. So, an experimental study to validate this previous work and demonstrate the possibility of increasing the printing velocity looking at modifications on cell viability might be done. The results of this study may help to improve μ-extrusion bioprinting and make it more competitive compared with inkjet or laser-assisted bioprinting, at least in terms of printing velocity.

Hence, the objective of this work is to experimentally study the movement accuracy of the bioprinter axes as well as the influence of volumetric flow, or printing speed attending to continuum equation, on cellular viability using a novel μ-extrusion bioprinter and a standard FDM 3D printing nozzle.

## 2. Materials and Methods

### 2.1. Bioprinter Design

**Figure 1:**
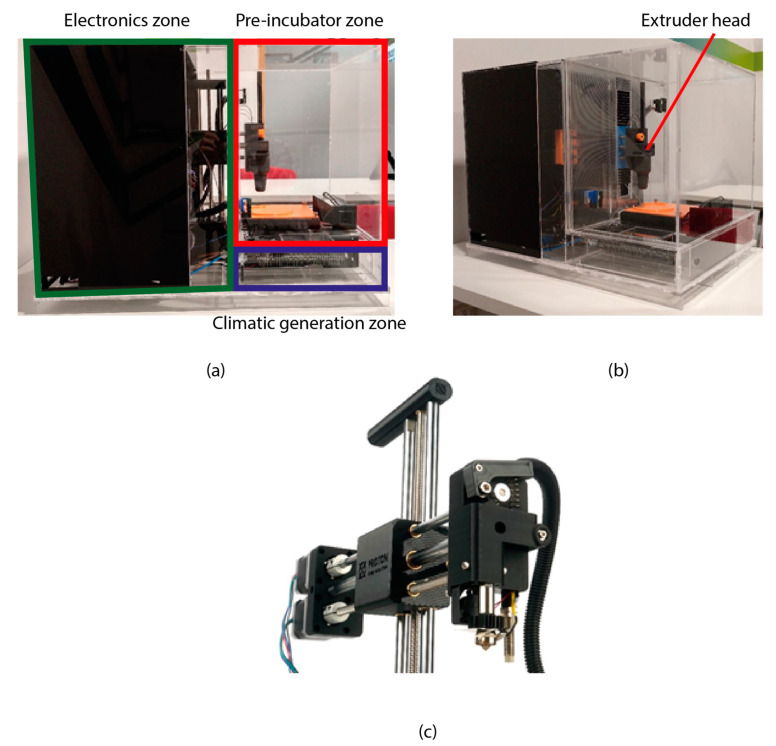
Bioprinter with pre-incubator system. (**a**) lateral view with the electronic (green), the climatic generator (blue) and the pre-incubator zone (red), (**b**) perspective view with the designed extruder head and (**c**) Atom Proton 3D printer extruder head.

The bioprinter used in this work was an adaptation of an Atom Proton 3D printer. (Atom 3D, Taipei, Taiwán) This 3D printer was selected because two main reasons, (I) it is a low-cost open-source 3D printer and (II) it has a different X axis construction. The differences between this Atom Proton and the Prusa type 3D printer (Figure 1) made the Atom Proton be the best choice to adapt a new extruder design and install it inside the pre-incubator. The design, components, construction and PID controller of the pre-incubator were detailed in a previous work [18] and shown in Figure 1a,b.

**Figure 2:**
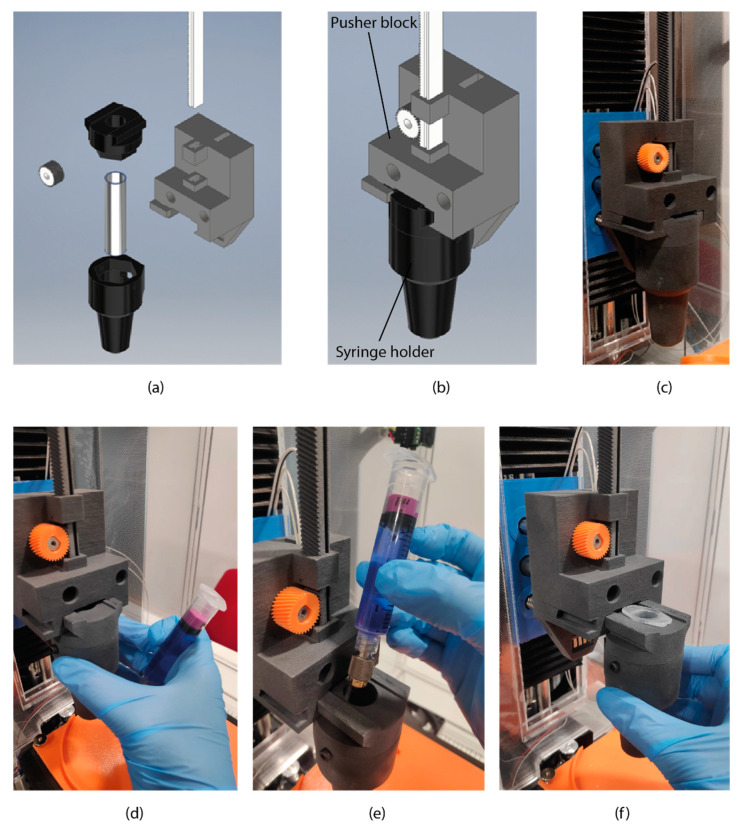
Designed extruder-head with rapid interchangeable syringe system. (**a**) exploit view of the system’s components, (**b**) full assembled system with indication of the pusher block and the syringe holder and (**c**) image of the prototype of the extrusion-head system. (**d**–**f**) operation of the quick interchangeable tool of a 5 mL syringe.

The new extruder head takes advantage of the location of the extruder motor and its power transmission. Figure 2a,b shows the two main parts of the new extruder design, the pusher block (white and grey parts) and the syringe holder (black parts). The piston-driven pusher block is formed by a rack and pinion system where the pinion is moved by the extruder motor causing the rack to move linearly up and down extruding the bioink. The syringe holder was a rapid interchangeable tool with non-contacts electronic pins to connect the 5 mL syringe heat block (100 kΩ thermistor and two flexible resistors) to the Atom Proton electronics. The bioink is placed in a 5 mL syringe with a specially designed plug to achieve uniform push. Figure 2d–f shows the operation of the rapid interchangeable tool loading a 5 mL syringe. Additionally, the specially designed plug and the proposed FDM 3D printing standard nozzle is shown in Figure 2e.

The firmware of the bioprinter was adapted from the original Atom Proton Marlin modifying the range of the axis, maximum and minimum operating temperatures and the axis steps per unit. Additionally, the bioprinter and the atmospheric enclosure were controlled by a Raspberry Pi 3 Model B (V3 Model B, Raspberry Pi Foundation, Cambridge, UK) through a custom-made interface similar to Repetier Host software.

### 2.2. Accuracy Measurements

**Figure 3:**
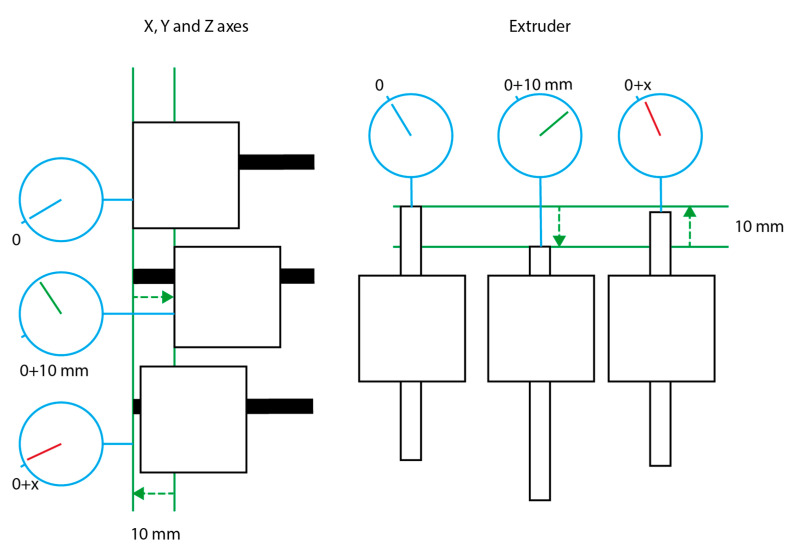
Schematic representation of the accuracy measurement protocol. X, Y and Z axes measurements (**left**) were done in 3 different points and extruder rack measurement (**right**) were done in a single point. All measurement were repeated 10 times.

Axis (X, Y and Z) and extruder linear movement accuracy was measured using a dial test indicator (Draper Expert N° 46609) with a 10 μm resolution. The tests were done in three measurement points for the X, Y and Z axis and in one measurement point for the extruder rack. The points were placed at zero (home), maximum axis length and at the middle of the axis. As the extruder did not have maximum and minimum position, one aleatory position of the rack was selected as the measurement point. The test consisted in positioning each axis at the selected point, moved the axis 10 mm and return to the initial position (Figure 3). The positioning error of each movement was repeated and measured 10 times for each selected point. The movement accuracy was calculated as the average value of all measurements for each axis. In a similar manner, the precision of the movement was calculated as the standard deviation of the previous accuracy. Additionally, the mean square error of the X-Y plane is calculated using the average value of each measurement point for X and Y axes obtaining a 3 × 3 matrix.

### 2.3. Cell Culture and Bioink Preparation

Human mesenchymal endometrial cells (endMSCs) were isolated from menstrual blood of a human donor. The study was conducted in accordance with the Declaration of Helsinki and the protocol was approved by the Ethics Committee of Minimally Invasive Surgery Centre (Project identification code: SITC215). All subjects gave their written informed consent to be included in the study [38] and were seeded at a density of 5000 cells/cm^2^ in T175 flasks incubated at 37 °C, 5% CO_2_ and 95% humidity and expanded in DMEM cell culture medium supplemented with 10% FBS (Gibco), 100 U/mL penicilin, 100 µg/mL streptomicin and 10 µg/mL cyprofloxacin. After approximately an 80% confluence was reached, the cells were trypsinized and resuspended in 1 mL of cell culture medium prior to mixing with the hydrogel.

The biomaterial used in the bioprinting tests was Cellink Bioink (Cellink, Gothenburg, Sweden). The final bioink mixture was done using a Cellink Mixer with a volumetric ratio of 5:1 and a final cell concentration of 7.5 × 10^5^ cells/mL.

### 2.4. Bioprinting

To study how different flow rate and nozzle affect cellular viability four different tests were performed. For each test, a specific G-code was programmed and the atmospheric enclosure was set to 37 °C and 50% of relative humidity. Each test consisted in the printing of a 40 mm × 40 mm scaffold in a Petri dish using a specific nozzle and flow rate. Table 1 summarizes the nozzle and flow rate used on each test as well as the printing velocity associated with the flow rate.

For the analysis, each printed scaffold was divided into 16 different parts using a scalpel. From all parts, eight were aleatory selected and placed in a 24 well plate to culture and measure cell viability.

### 2.5. Cell Viability Evaluation Assay

Samples from each test were cultured and tested for cell viability at 0 (D0), 1 (D1), 3 (D3) and 7 (D7) days after the bioprinting procedure. Cellular viability of each sample was analyzed using Invitrogen Live/Dead kit (Invitrogen™ R37601, Thermo Fisher Scientific, Waltham, MA, USA), staining live cells in green (488 nm) and dead cells in red (570 nm).

The cell viability images were obtained using a fluorescence microscope (Nikon TE2000-S, Tokyo, Japan) and cell viability was quantified using open-source ImageJ 1.52p software (National Institutes of Health, Bethesda, Rockville, MD, USA) by converting live and dead cells images of each test to 8-bit grayscales images, adjusting the histogram to get brighter images and reducing the background noise using the subtracting background process. Then, a composite image was obtained using the previous adjusted images. Finally, live and dead cells were manually counted to obtain the cell viability calculated as: live/(live + dead).

Cell viability results were processed using Graphpad Prism v. 9.1.0 (Graphpad Software, San Diego, CA, USA) using a two-way ANOVA with Tukey’s multiple comparison test, with 95% confidence interval (significance threshold *p* = 0.05).

## 3. Results and Discussion

### 3.1. Bioprinter Accuracy

Table 2 shows the absolute errors obtained from accuracy test for X, Y and Z axis and Extruder.

**Figure 4:**
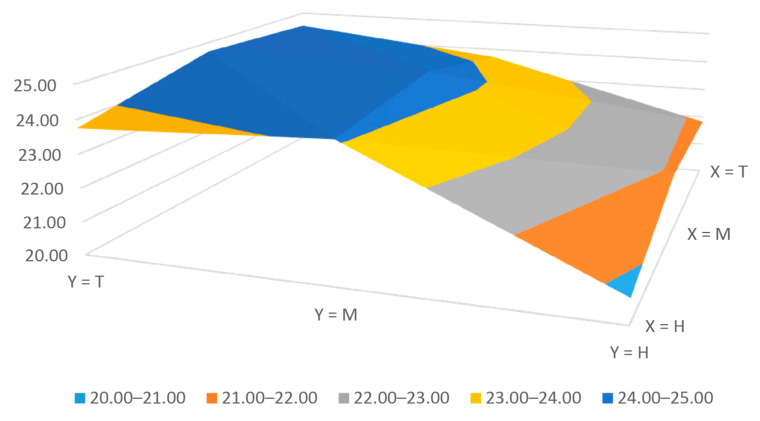
Mean square error of the X-Y plane in μm.

The average absolute errors or the movement accuracy and precision obtained are 11.67 ± 10.85, 16.33 ± 11.89 and 8.67 ± 10.08 μm for X, Y and Z axis, respectively. Additionally, the movement accuracy and precision of the extruder rack is 5.00 ± 7.07 μm. Therefore, the adapted Atom Proton bioprinter has a X, Y and Z movement accuracy under 17 μm with a movement precision around 12 μm. The bioprinter also has a new extruder system capable of extruding bioinks with a linear rack movement accuracy of 5 μm and a precision around 7 μm. Additionally, the mean square error for the X-Y plane can be seen in Figure 4. This error indicates the possible movement deviation of the extruder head in the X-Y plane. The distribution of the error can be divided into two zones, a plateau zone (from middle to total length of the Y axis) and a ramp from the lowest error values (home in Y axis) to the middle line of the X-Y plane. Despite, the plateau zone has higher error values, the difference between the maximum and minimum error is 0.98 μm so it can be considered a stable area for printing with an average error in the X-Y plane of 24.13 μm. Additionally, the total error variation in the plane is 3.98 μm. All X-Y plane errors can be considered negligible in many bioprinting applications because the width of the extruded line is usually higher of 200 μm (27G conical tip or needle). All these errors might be due to the different axes’ movements compared with the standard Prusa type printers. While the Atom Proton axes’ movements are looser guided because it only has one z motor, the Prusa has two, so the extruder head might quiver more in our setup.

Comparing our movement precision with other bioprinters, we have obtained similar movement precision than other authors. Table 3 shows the axes movement accuracy of the proposed bioprinter and the custom made μ-extrusion bioprinters. Specifically, Kahl et al. [23], Reid et al. [24] and Hesuani et al. [39] obtained a X-Y movement precision of 12 μm, 13 μm and 10 μm, respectively while Z movement precision obtained by Kahl et al. [23] is 4 μm. Regarding commercial bioprinters, Ozbolat et al. [14] summarized this information from several bioprinters and obtained position precision varying between 5 and 150 μm of BioBot 1 (BioBots/Allevi, Philadelphia, PA, USA) and Regemat 3D V (Regemat 3D, Granada, Spain), respectively.

**Figure 5:**
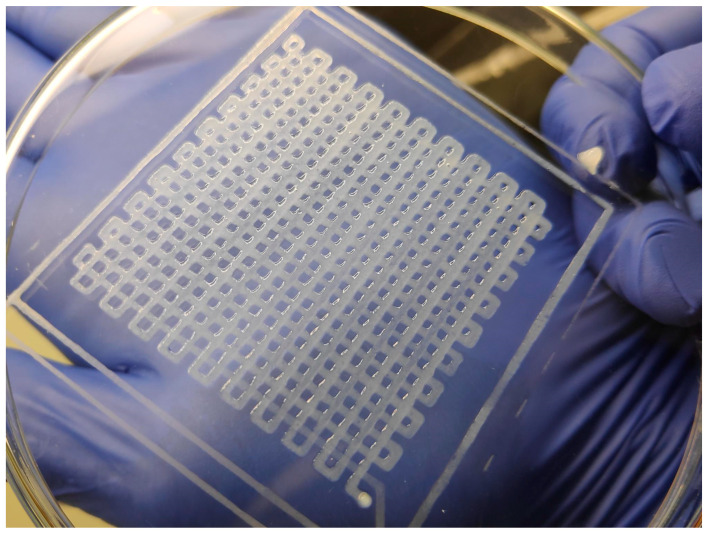
Printed scaffold with the maximum volumetric flow and printing velocity in the study (T4 test).

Regarding to the printed structures, Figure 5 shows the scaffold obtained in T4 test. It can be seen that the scaffold is well shaped with uniform distribution of lines and holes. Nevertheless, differents line width can also be seen. The point in the bottom part of the figure corresponds with the home point. In the printing procedure the movement of the extruder head accumulated errors and the line width varies with correspondence on Figure 4.

### 3.2. Cell Viability

**Figure 6:**
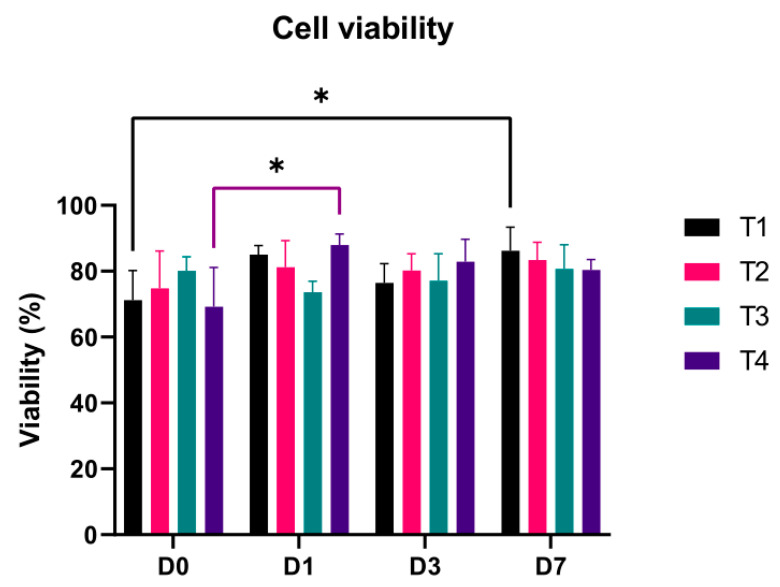
Cell viability of test grouped by timepoints. * represents significative difference (*p* < 0.05).

Figure 6 shows the results of cell viability for all tests grouped by timepoints. Results show that the proposed use of a standard 3D printing nozzle is as good as the commonly used conical tip, as no significant differences between printing conditions can be found within the same timepoint (Figure 6). Furthermore, cell viability of the conical tip (T1) and the faster nozzle (T4) are similar independently of the measured days. Specifically, T1 and T4 cell viability values are 71.22 and 69.22%, 85.05 and 87.93%, 76.47 and 82.88% and 86.20 and 80.34% for D0, D1, D3 and D7, respectively. Additionally, the maximum and minimum values of cell viability are 69.22 and 80.15% for D0, 73.62 and 87.93% for D1, 76.47 and 82.88% for D3 and 80.34 and 86.20% for D7.

**Figure 7:**
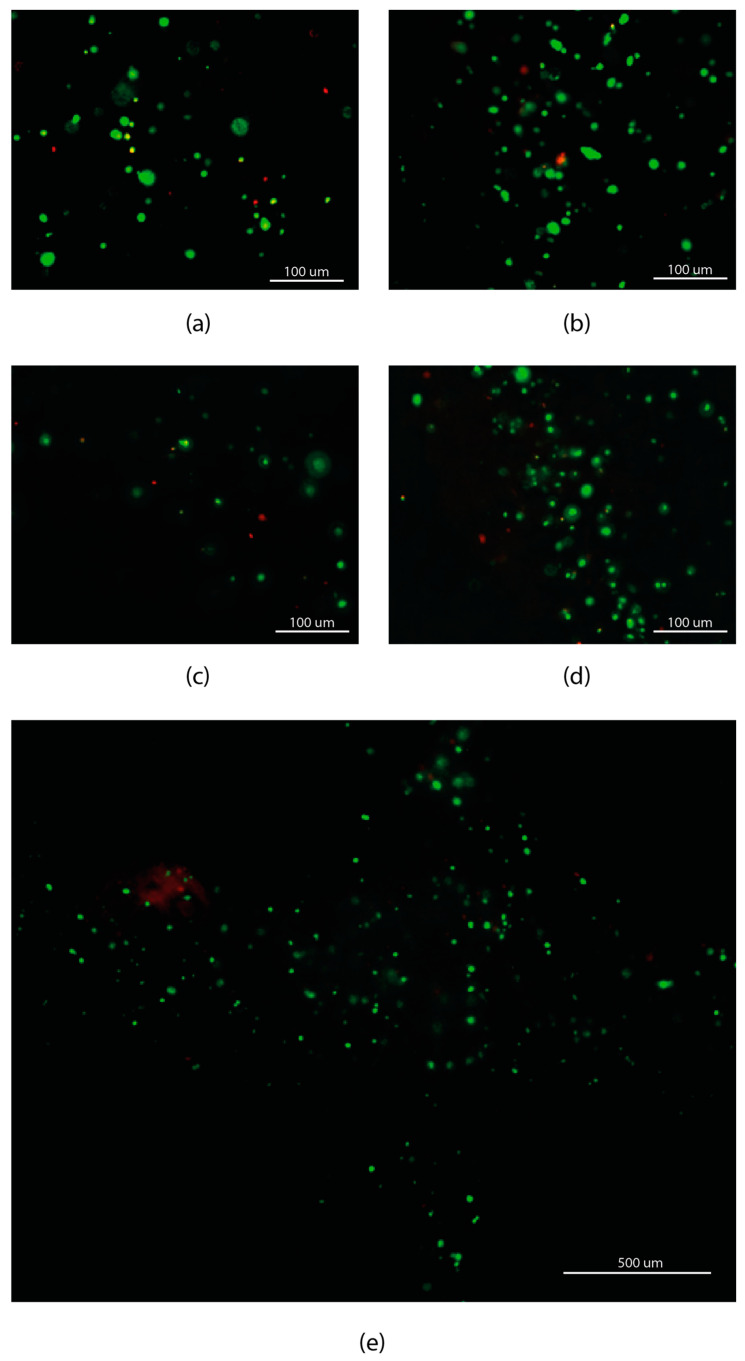
Cell viability images from samples with significance differences. (**a**) Cell viability of T1 measured at day 0, (**b**) cell viability of T1 measured at day 7, (**c**) cell viability of T4 measured at day 0 and (**d**) cell viability of T4 measured at day 1. Cells distribution of T4 at day 7 (**e**).

Looking at the evolution of cell viability over time, an increasing tendency of the minimum value can be seen. In this study, there are not significant differences for T2 and T3, independently of the day, but significant differences between D0 and D4 and D0 and D1 can be seen for T1 and T4, respectively (Figure 7). On the one hand, the differences of T1 can be explained as the cell proliferation when the scaffolds samples are cultured. Cell viability of T1 tends to increase with culture time. On the other hand, the different cell viability of T4 from day 0 to day 1 can be explained as both an acclimatization process to the encapsulation and an increase in cell proliferation due to the continuation of the cell cycle. Before trypsinization, cells can be at any of the cell cycle phases and after encapsulation in the hydrogel they resume the cell cycle before becoming dormant, thus causing an increase in proliferation. Another possible explanation could be the healing process of those cells that might be injured during the bioprinting procedure (Figure 7). The healing or recovery process of cells in bioprinting procedures have been studied by authors as Ozbolat et al. [4] or Ning et al. [40]. In their works, Ozbolat obtained a cell viability increment from 43.92 to 76.06% in two days culture caused by cell healing as well as cell proliferation, while Ning obtained a cell recovery of approximately 2.5% in 6 h after printing. Additionally, Figure 7e shows the uniform spatial distribution of the cells within the printed scaffold.

Additionally, the range of values for each day tend to reduce as result of cell healing and proliferation [4,40] when samples are cultured for several days in alginate hydrogels. Nevertheless, other authors as Schmidt et al. [41] obtained negligible cell proliferation rate even after 14 days of culture on Cellink Bioink. Longer culture periods would also allow endMSCs to evolve from a spherical shape when encapsulated inside the hydrogel to a shape with short branches and more expanded geometry [41]. Hence, further research on this topic will be accomplished in future works.

Therefore, according to our results, the use of the proposed FDM standard 3D printing nozzle does not produce a noticeable harmful effect to cells when printing twice faster than with the conical tip.

Cell viability results obtained are similar to those of other authors testing new bioprinters or adapted print-heads (Table 4). Specifically, Campbell et al. [25] calculate the relative cell viability of their samples with respect to their hand bioprint measured 24 h after the procedure. They do not provide any value of cell viability to compare with our results, but in the graph, they presented the cell viability tend to decrease. Bessler et al. [21] studied mESC and HEK293 cells using a FRESH printing method and they obtained cell viability varying from 60 to 95%. Specifically, they measured viability just after the printing procedure and obtained 85 ± 13% and 81 ± 9% for mESC and HEK293 cells, respectively. Kahl et al. [23] studied HEK 293 cell viability but they presented their results as the average relative fluorescence intensity (RFU) instead the cellular viability itself. They concluded that as their RFU increase with culture time the cell proliferation and viability of the cultured sample increase. Ozbolat et al. [4] obtained cell viabilities of bioprinted filaments and spheroids using Cartilage progenitor cells (CPCs). In their work, they reported a cell viability at day 1 and 7 that increases from 43.92 to 87.23% and 60.15 to 92.87% for filaments and spheroids, respectively. They also explained the increment in cellular viability as the results of cell healing and proliferation while culture. Roehm et al. [42] studied how different flow rate and print velocity through a nozzle affect cellular viability measured 5 days after the printing procedure. They obtained similar results to ours with cell viabilities approximately ranged between 60 to 80% and none of the flow rates or velocities they checked had a significant influence on cellular viability. Sanz-García et al. [43] obtained higher cell viability values than ours (approximately 90%). They used hASCs cells in a 10% Gelatin-2%Alginate bioink and a new pneumatic extrusion-head with a peltier cooling/heating system and a 25G conical tip.

## 4. Conclusions

In this work, a new bioprinter has been designed and tested. Our results show that the new bioprinter has a movement accuracy under 17 μm with a movement precision of approximately 12 μm for the X, Y and Z axes. Moreover, the rack, located in the piston-driven print-head pusher block, was demonstrate to be capable of extruding material with an accuracy under 5 μm and a precision around 7 μm. Additionally, as the proposed use of a standard FDM 3D printing nozzle brings theoretical higher volumetric flow, the tests performed in this work show that by using this nozzle it is possible to obtain up to double faster printings without compromising cell viability. Specifically, cell viability increments from 70% just after printing to 90% at day 7, as result of cell healing and proliferation. Therefore, the proposed bioprinter with its new extrusion-head and a standard FDM 3D printing nozzle allows to control the atmospheric temperature and humidity performing faster printings while maintain high cell viability. Future studies should explore the capacity of the printer to produce larger or more complex scaffolds, requiring longer printing times, while maintaining high cell viability.

In future works, a more comprehensive study to obtain the maximum velocity and flow rate of the FDM 3D printing standard nozzle without compromising cell viability must be done.

## Figures and Tables

**Table 1:** Nozzle, flow rate and associated printing velocity of the different test performed.

Test	Nozzle	Flow Rate (mm^3^/s)	Printing Velocity (mm/s)
T1	22G conical tip	10	8.55
T2	E3D v6 standard nozzle	10	8.55
T3	E3D v6 standard nozzle	15	12.83
T4	E3D v6 standard nozzle	20	17.11

**Table 2:** Absolute errors in μm of the X, Y, Z axis and the extruder rack. Initial or Home (H), Middle (M) and Total (T) length measurements points.

Sample	X Error (μm)			Y Error (μm)			Z Error (μm)			Extruder Error (μm)
	H	M	T	H	M	T	H	M	T	
1	30	40	20	10	30	20	10	20	10	20
2	0	0	0	10	40	10	10	30	10	0
3	20	10	20	20	40	30	10	40	10	0
4	0	10	0	20	30	10	10	20	20	0
5	20	10	10	0	0	20	0	10	20	0
6	20	20	20	0	0	10	0	0	0	0
7	20	10	20	20	0	10	0	10	10	10
8	0	20	0	20	10	20	0	0	0	10
9	0	10	20	20	0	30	0	10	0	10
10	0	20	0	20	10	30	0	0	0	0

**Table 3:** Movement precision (μm) of designed bioprinter and bibliography custom made μ-extrusion bioprinters.

Axis	Studied Bioprinter	Khal et al. [23]	Reid et al. [24]	Hesuani et al. [39]
X	11.67	12.00	13.00	10.00
Y	10.85	12.00	13.00	10.00
Z	11.98	4.00	-	-
Extruder	5.00	-	-	-

**Table 4:** Measurement time, cell line and cell viability obtained using different hand-made μ-extrusion bioprinters.

Hand-Made Bioprinters	Measurement Time	Cell Line	Cell Viability
New extruder	0, 1, 3 and 7 day	endMSCs	69.20 to 87. 93%
Campbell et al. [25]	1 day	HUVECs	Decreased in time
Bessler et al. [21]	0 day	mESC and HEK293	60 to 95%
Kahl et al. [23]	0, 2, 5 and 7 day	HEK 293	RFU–increased in time
Ozbolat et al. [4]	1 and 7 day	CPCs	43.92 to 92.87%
Roehm et al. [42]	5 day	IMR-32	60 to 80%
Sanz-García et al. [43]	0 and 1 day	hASCs	90%

## Data Availability

The data are not publicly available.
